# Social Fear Affects Limbic System Neuronal Activity and Gene Expression

**DOI:** 10.3390/ijms23158228

**Published:** 2022-07-26

**Authors:** Catharina S. Hamann, Julian Bankmann, Hanna Mora Maza, Johannes Kornhuber, Iulia Zoicas, Angelika Schmitt-Böhrer

**Affiliations:** 1 Center of Mental Health, Department of Psychiatry, Psychosomatics and Psychotherapy, University of Würzburg, 97080 Würzburg, Germany; catharina.hamann@gmx.de (C.S.H.); julian.bankmann@posteo.de (J.B.); hannamora.hm@gmail.com (H.M.M.); 2 Department of Psychiatry and Psychotherapy, Friedrich-Alexander University Erlangen-Nürnberg (FAU), 91054 Erlangen, Germany; johannes.kornhuber@uk-erlangen.de (J.K.); iulia.zoicas@uk-erlangen.de (I.Z.)

**Keywords:** social anxiety, fear expression, social avoidance, gene expression, Npy, Npyr1, Npyr2, Htr1a, Htr2a, Htr2c

## Abstract

Social anxiety disorder (SAD) is a highly prevalent and comorbid anxiety disorder with rather unclear underlying mechanisms. Here, we aimed to characterize neurobiological changes occurring in mice expressing symptoms of social fear and to identify possible therapeutic targets for SAD. Social fear was induced via social fear conditioning (SFC), a validated animal model of SAD. We assessed the expression levels of the immediate early genes (IEGs) cFos, Fosl2 and Arc as markers of neuronal activity and the expression levels of several genes of the GABAergic, serotoninergic, oxytocinergic, vasopressinergic and neuropeptide Y (NPY)-ergic systems in brain regions involved in social behavior or fear-related behavior in SFC+ and SFC− mice 2 h after exposure to a conspecific. SFC+ mice showed a decreased number and density of cFos-positive cells and decreased expression levels of IEGs in the dorsal hippocampus. SFC+ mice also showed alterations in the expression of NPY and serotonin system-related genes in the paraventricular nucleus of the hypothalamus, basolateral amygdala, septum and dorsal raphe nucleus, but not in the dorsal hippocampus. Our results describe neuronal alterations occurring during the expression of social fear and identify the NPY and serotonergic systems as possible targets in the treatment of SAD.

## 1. Introduction

Among the wide spectrum of anxiety disorders, social anxiety disorder (SAD) is the second most common disorder with a lifetime prevalence of around 12% [1] and a 12-month prevalence of around 2.3% [2]. According to the Diagnostic and Statistical Manual of Mental Disorders (DSM)-V, the core symptoms of SAD include a profound fear and avoidance of social situations, where the person feels observed or scrutinized by others. The immense fear often leads to withdrawal from social situations and sometimes complete avoidance of social situations [3]. This can have debilitating consequences for the living conditions and working circumstances of affected individuals [4]. Known comorbid diseases include other anxiety disorders, depression and substance use disorders [5], with the majority of SAD patients reporting at least one other psychiatric disorder [6] and about 25% of the patients reporting three or more psychiatric disorders [7]. In most cases, SAD has an earlier onset, suggesting that SAD is a risk factor for the development of additional psychiatric disorders [6]. Early diagnosis and intervention might therefore reduce the risk of developing additional psychiatric disorders and might facilitate an improved treatment outcome. State of the art therapy includes cognitive behavioral therapy [8] and pharmacotherapy [9], where the use of selective serotonin reuptake inhibitors (SSRIs) delivers the best response rates [9]. However, many patients do not respond to the existing therapies [6,9,10], which highlights the need for alternative treatment options, especially in the area of new pharmaceutical approaches. 

Despite intense research, the mechanisms behind social anxiety and fear and the underlying neural circuitries are not yet completely understood [11]. Studies in SAD patients reported increased activity of the amygdala [12,13,14,15], an almond-shaped group of nuclei involved in the perception, learning and expression of fear [16], which might explain the exaggerated fear responses observed in these patients. Other studies described altered activity of the hippocampus in SAD patients [17,18]. The hippocampus is a brain region involved in declarative memory [19], that also processes information about the context, i.e., environment of a fearful situation [20]. Preclinical studies made use of several stress paradigms implying either physical stressors, social stressors or a combination of both, to model SAD in rodents [21,22]. Most of these paradigms, however, also induced comorbidities in measures of depressive-like behavior, generalized-anxiety-like behavior and substance abuse and did not allow for the study of social fear in particular [21,22]. A relatively new animal model, the social fear conditioning (SFC) paradigm, was shown to induce specific social fear and avoidance of conspecifics without comorbidities in measures of depressive-like behavior, generalized-anxiety-like behavior and alcohol consumption [23,24]. The socially fearful phenotype could be reversed by chronic administration of the SSRI paroxetine and of the tricyclic antidepressant amitriptyline, demonstrating the predictive validity of the SFC model [23,25]. Similar to SAD patients after exposure to a social stressor [26,27], socially fear conditioned (SFC+) mice showed increased corticosterone levels after exposure to a social stimulus [24]. These findings suggest that at least parts of the mechanisms and neural circuitries underlying social fear might be similar between SFC+ mice and SAD patients.

In this study, we aimed to characterize some of the neurobiological changes occurring during the expression of social fear, which might help to identify possible therapeutic targets for SAD. We investigated the neuronal activity and the expression level of genes of several neurotransmitter and neuropeptide systems in brain regions known to be involved in social behavior and/or fear-related behavior [28,29,30,31] in mice expressing symptoms of social fear. For this, we exposed SFC+ and unconditioned control (SFC−) mice to an unfamiliar conspecific (i.e., social stimulus) and collected brain tissue 2 h later. We assessed the expression levels of three different immediate early genes (IEG), i.e., cFos, Fosl2 and Arc, as markers of neuronal activity [32,33,34] and the expression levels of several genes of the gamma-aminobutyric acid (GABA), serotonin, oxytocin (OXT), vasopressin (AVP) and neuropeptide Y (NPY) systems. An involvement of the GABA-ergic and serotonergic systems in the expression of social fear is suggested by the fact that benzodiazepines and antidepressants that act on these systems reduced the expression of SFC-induced social fear [23,25]. Pharmacological studies also indicate an involvement of neuropeptide systems in the expression of social fear. For example, intracerebroventricular (i.c.v.) administration of OXT reduced the expression of SFC-induced social fear via activation of the OXT receptor (OXTR) [35]. Similarly, i.c.v. administration of NPY reduced the expression of SFC-induced social fear via simultaneous activation of NPY receptors 1 (NPYR1) and NPYR2 [36,37]. Given that a decreased neurogenesis was described in several models of social stress [38,39] and in mice showing social avoidance [40,41], we also investigated the expression of neurogenesis-related genes. We describe neuronal alterations occurring during the expression of social fear and identify the dorsal hippocampus as an important brain region for the expression of social fear. We also identify the NPY and serotonergic system as possible targets in the treatment of SAD. 

## 2. Results

### 2.1. Behavioral Analyses Confirm Successful Social Fear Conditioning

**Figure 1:**
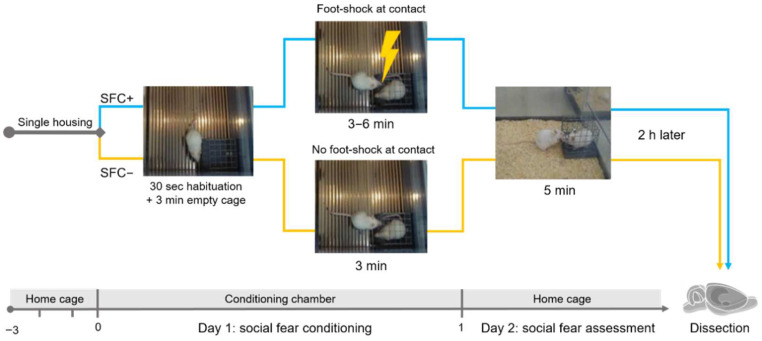
Schematic representation of the experimental paradigm. Mice were single-housed for three days before the experiment started. On day 1, during social fear conditioning (SFC), the mice were placed in the conditioning chamber and, after a 30 s habituation period, were exposed to an empty wire mesh cage to investigate the pre-conditioning non-social anxiety. After 3 min, this non-social stimulus was replaced with an identical cage containing an unfamiliar male mouse. Unconditioned control (SFC−) mice were allowed to investigate this social stimulus for 3 min, whereas conditioned (SFC+) mice received a mild foot shock (0.7 mA, 1 s) each time they investigated, i.e., made direct contact with the social stimulus. On day 2, SFC− and SFC+ mice were exposed for 5 min in their home cage to an unfamiliar social stimulus to assess social investigation as a parameter of social fear. After 2 h, brains were collected for further processing.

To induce social fear, SFC+ mice received during SFC on day 1 mild electric foot shocks each time they investigated an unfamiliar conspecific (i.e., social stimulus), whereas SFC− mice investigated a social stimulus without receiving foot shocks [23,24,35,36,42,43,44,45]. One day after SFC, the time that the SFC+ and SFC− mice spent investigating an unfamiliar social stimulus was assessed as a readout of social fear (Figure 1).

**Figure 2:**
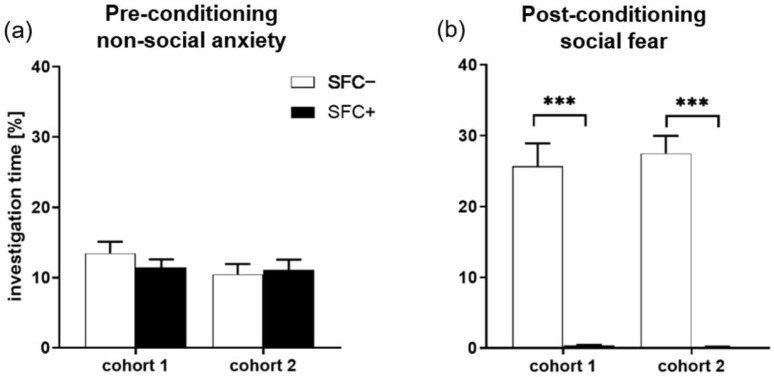
Social fear conditioning (SFC) induces social fear. (**a**) Pre-conditioning investigation of the non-social stimulus (empty cage) during SFC on day 1 shown by the unconditioned control (SFC−) mice and conditioned socially fearful (SFC+) mice. Cohort 1 represents the mice used for the immunohistochemistry study (*n* = 9 SFC−; *n* = 10 SFC+), whereas cohort 2 represents the mice used for the gene expression study (*n* = 10 SFC−; *n* = 10 SFC+). (**b**) Investigation of the social stimulus during social fear assessment on day 2. Data represent means + SEM. Statistical analysis was performed using a two-sample comparison test; *p* *** < 0.001.

The results of the behavioral investigations demonstrate that SFC induced social fear (Figure 2b, Table S1). Two different mouse cohorts were used for this study. Cohort 1 refers to the mice used for the immunohistochemistry (IHC)/immunofluorescence staining study, and cohort 2 refers to the mice used for the gene expression analysis. 

During SFC on day 1, all mice spent a similar amount of time investigating the non-social stimulus (empty cage) (Figure 2a), which indicates similar pre-conditioning non-social anxiety levels between SFC− and SFC+ mice. During social fear assessment on day 2, SFC+ mice spent less time investigating the social stimulus (unfamiliar conspecific) compared to respective SFC− mice, indicating increased social fear (Figure 2b, cohort 1: T(17) = −17.142; *p* < 0.001; cohort 2: T(18) = −11.125; *p* < 0.001). SFC+ but not SFC− mice also showed attempt approaches and freezing, indicating increased fear responses (Table S1). SFC− and SFC+ mice showed a similar number of total line crossings during social fear assessment on day 2, indicating comparable levels of locomotor activity (Table S1). However, SFC− mice showed more proximal line crossings (Table S1, cohort 1: T(17) = 7.160; *p* < 0.001; cohort 2: T(18) = 6.907; *p* < 0.001) and less distal line crossings (cohort 1: T(17) = −3.791; *p* = 0.001; cohort 2: T(18) = −3.876; *p* = 0.001) compared with SFC+ mice. This suggests that SFC− mice moved more around the social stimulus, corresponding to their increased social investigation, whereas SFC+ mice moved more at the opposite side of the cage, corresponding to their socially fearful and avoidant phenotype.

### 2.2. Social Fear Decreases Neuronal Activity in the Dorsal Dentate Gyrus, but Not in Other Dorsal Hippocampus Regions or in the Ventral Hippocampus

**Figure 3:**
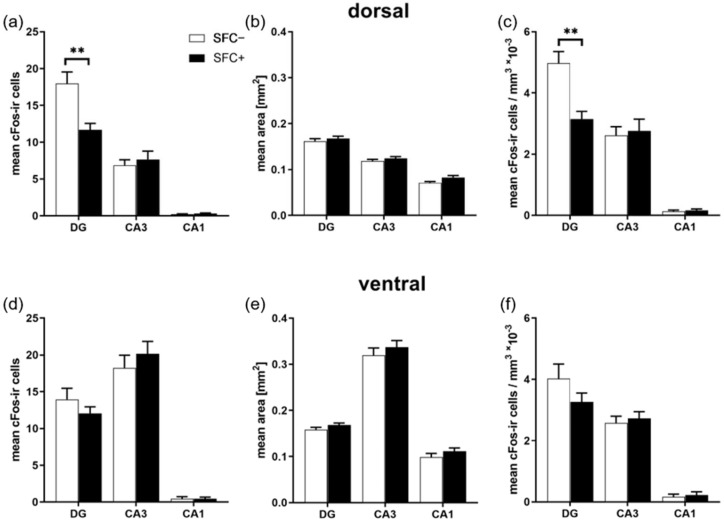
Social fear expression reduces the number and density of cFos-positive neurons exclusively in the dorsal hippocampus. (**a**,**d**) Mean number of cFos-ir cells in examined sections within the (**a**) dorsal and (**d**) ventral hippocampus of the granular cell layer of the dentate gyrus (DG), and the pyramidal cell layers of the cornu ammonis area 3 (CA3) and 1 (CA1). (**b**,**e**) Mean area of investigated layers in mm^2^. (**c**,**f**) Mean cell density (mean number of cFos-ir cells/mm^3^). SFC−, unconditioned control mice (*n* = 9); SFC+, conditioned socially fearful mice (*n* = 10). Data represent means + SEM. Statistical analysis was performed using a two-sample comparison test; *p* ** < 0.01.

In order to investigate the neuronal activity during the expression of social fear, brain tissue was collected 2 h after exposure to the social stimulus during social fear assessment (Figure 1). A quantitative IHC study with an antibody detecting the IEG cFos was performed to investigate neuronal activity in the dorsal and ventral hippocampus. Quantification of cFos-immunoreactive (cFos-ir) cells revealed a lower number and density of cFos-positive cells in the granular cell layer (gcl) of the dentate gyrus (DG) of the dorsal hippocampus of SFC+ mice compared to SFC− mice (Figure 3a: T(17) = −3.305; *p* = 0.004; Figure 3c, T(17) = −3.796; *p* = 0.004, Table S2). The pyramidal cell layers (pcl) of the cornu ammonis 3 (CA3) and 1 (CA1) of the dorsal hippocampus as well as the gcl of the DG and the pcl of CA3 and CA1 of the ventral hippocampus revealed no changes in the number and density of cFos-ir cells (Table S2).

### 2.3. Social Fear Does Not Affect Neuronal Activity in the PVN

In order to investigate the distribution of cFos-positive cells in the paraventricular nucleus of the hypothalamus (PVN), an immunofluorescence double staining study with an antibody detecting the IEG cFos and an immunofluorescent AVP antibody was performed. The AVP antibody was used as a form of counterstaining to determine the precise area of the PVN. Quantification of the number of cFos-ir cells (Figure S1a), as well as investigation of mean area (Figure S1b) and density of cFos-ir cells (Figure S1c) showed no differences between SFC+ and SFC− mice in the PVN (Table S3).

### 2.4. Social Fear Reduces the Expression Levels of Immediate Early Genes in the Dorsal Hippocampus

**Figure 4:**
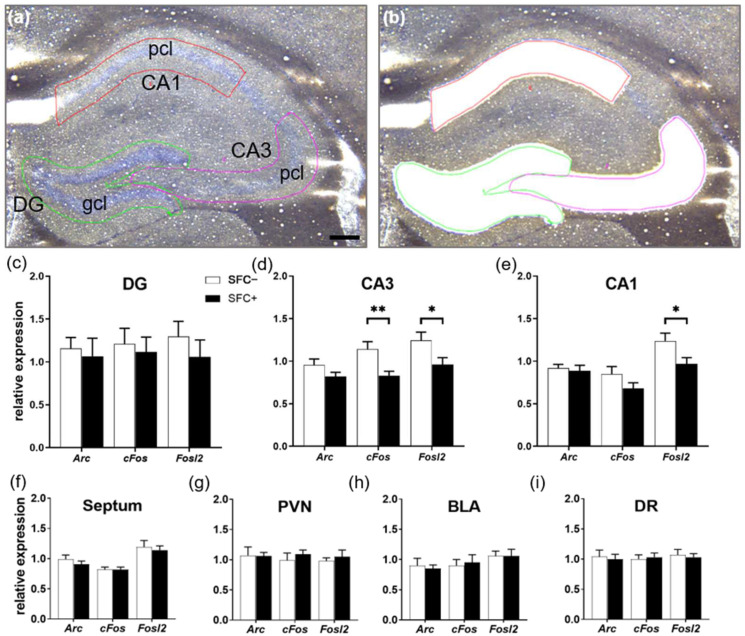
Social fear reduces the expression levels of immediate early genes exclusively in the pyramidal cell layer (pcl) of the cornu ammonis area 3 (CA3) and 1 (CA1) of the dorsal hippocampus. (**a**) Representative cresyl violet staining of the dorsal hippocampus before and (**b**) after sequential dissection with laser capture microdissection. Areas depicted are the granular cell layer (gcl) of the dentate gyrus (DG) and the pcl of CA3 and CA1. (**c**–**i**) Relative expression levels of the immediate early genes *Arc*, *cFos* and *Fosl2* in seven different target regions: (**c**) the gcl of DG, (**d**) the pcl of CA3 and (**e**) CA1, (**f**) the septum, (**g**) the paraventricular nucleus of the hypothalamus (PVN), (**h**) the basolateral amygdala (BLA) and (**i**) the dorsal raphe nucleus (DR). SFC−, unconditioned control mice (*n* = 10); SFC+, conditioned socially fearful mice (*n* = 10). Scale bar in (a) represents 200 µm. Data represent means + SEM. Statistical analysis was performed using a two-sample comparison test. *p* * < 0.05; *p* ** < 0.01.

To investigate whether the reduced number and lower density of cFos-ir cells found in the dorsal DG in SFC+ mice is replicated by decreased cFos mRNA levels, we assessed gene expression levels of *cFos* as well as of two other IEGs, *Arc* and *Fosl2* (Table S4), in the dorsal hippocampus of SFC+ and SFC− mice. As no differences in the cFos protein were found in the ventral hippocampus, gene expression was not investigated in this brain region. RNA was extracted from tissue cut from tissue sections containing the dorsal hippocampus using laser capture microdissection (Figure 4a,b), which allowed the precise extraction of specific brain regions up to small sub-regions and cell layers. As depicted in Figure 4, SFC+ mice showed reduced expression levels of *cFos* (T(18) = −3.099; *p* = 0.006) and *Fosl2* (T(18) = −2.257; *p* = 0.037) in the pcl of CA3 (Figure 4d) and reduced expression levels of *Fosl2* in the pcl of CA1 (Figure 4e; T(18) = −2.320; *p* = 0.032). 

Similar expression levels of IEGs between SFC+ and SFC− mice were found in the gcl of the DG (Figure 4c), septum (Figure 4f), PVN (Figure 4g), basolateral amygdala (BLA) (Figure 4h), as well as in the dorsal raphe nucleus (DR) (Figure 4i). 

### 2.5. Social Fear Alters the Expression of Neuropeptide Y and Serotonin System-Related Genes

To investigate whether the expression of social fear is accompanied by alterations at the level of several neurotransmitter and neuropeptide systems involved in social behavior and/or fear-related behavior [23,25,28,29,30,31,35,36,37], we assessed the expression level of genes of the GABA, serotonin, OXT, AVP and NPY systems in the dorsal hippocampus, septum, PVN, BLA and DR of SFC+ and SFC− mice. We also investigated the expression of neurogenesis-related genes, given that a decreased neurogenesis was described in mice showing social avoidance [40,41].

**Figure 5:**
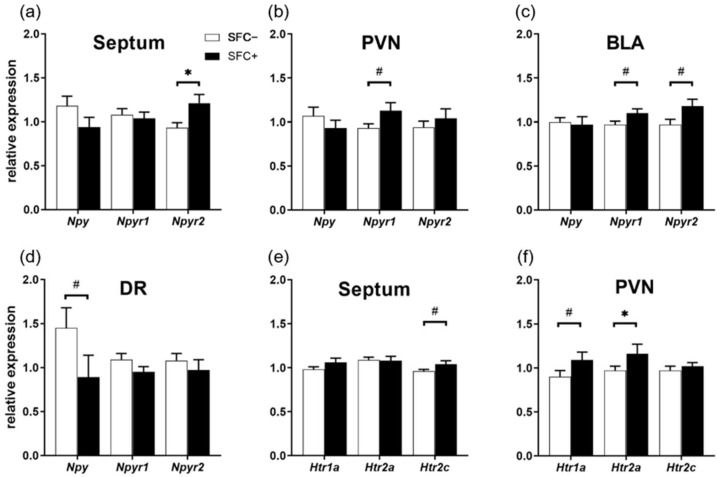
Social fear alters the expression of neuropeptide Y and serotonin-system-related genes. (**a**–**d**) Relative expression levels of neuropeptide Y (*Npy*), Npy receptor 1 (*Npyr1*) and 2 (*Npyr2*) genes in (**a**) the septum, (**b**) the paraventricular nucleus (PVN) of the hypothalamus, (**c**) the basolateral amygdala (BLA) and (**d**) the dorsal raphe nucleus (DR). (**e**,**f**) Relative expression levels of serotonin receptor (*Htr)1a*, *Htr2a* and *Htr2c* genes in the (**e**) septum and (**f**) PVN. SFC−, unconditioned control mice (*n* = 10); SFC+, conditioned socially fearful mice (*n* = 10). Data represent means + SEM. Statistical analysis was performed using a two-sample comparison test; *p* ^#^ < 0.1; *p* * < 0.05.

Brain-region-dependent differences between SFC+ and SFC− mice were found in the quantitative estimation of mRNA levels of NPY system-related genes such as *Npy* and its two analyzed receptors *Npyr1* and *Npyr2*. As such, SFC+ mice showed a higher expression of *Npyr2* in the septum (Figure 5a, T(18) = 2.482; *p* = 0.035) and a trend towards a higher expression of *Npyr1* in the PVN (Figure 5b, T(18) = −1.977; *p* = 0.064) and the BLA (Figure 5c, T(18) = −1.933; *p* = 0.069), and of *Npyr2* in the BLA (Figure 5c, T(18) = −1.894; *p* = 0.074). SFC+ mice also showed a trend towards a lower expression of *Npy* in the DR (Figure 5d, T(16) = 1.636; *p* = 0.077). The expression of *Npy*, *Npyr1* and *Npyr2* genes was not different between SFC+ and SFC− mice in the DG, CA3 and CA1 regions of the dorsal hippocampus (Table S4).

Quantitative estimation of mRNA levels of three different receptors of the serotonergic system (i.e., *Htr1a*, *Htr2a* and *Htr2c*) also revealed differences between SFC+ and SFC− mice. SFC+ mice showed a higher expression of *Htr2a* in the PVN (Figure 5f, T(18) = −1.562; *p* = 0.023), and a trend towards higher expression of *Htr2c* in the septum (Figure 5e, T(18) = −1.860; *p* = 0.079) and of *Htr1a* within the PVN (Figure 5f, T(18) = −1.547; *p* = 0.089). Similar to the expression of NPY-system-related genes, gene expression of the analyzed serotonin receptors was not changed in the DG, CA3 and CA1 regions of the dorsal hippocampus.

Evaluation of the expression of adult neurogenesis-related genes (*Dcx* and *Mcm2*), of genes related to the GABAergic system (*Gad1*), AVP system (*Avp, Avpr1a*) and OXT system (*Oxt, Oxtr*), did not show any significant differences between SFC+ and SFC− mice in the analyzed brain regions. This suggests that these neurotransmitter and neuropeptide systems within the selected brain regions might not be related to the expression of social fear (Table S4).

## 3. Discussion

The present study demonstrates that social fear reduces neuronal activity at the level of the dorsal hippocampus but not at the level of the ventral hippocampus nor in other brain regions such as the septum, PVN, BLA and DR. Conversely, SFC+ mice show alterations in the expression of NPY system- and serotonin system-related genes in the septum, PVN, BLA and DR but not in the dorsal hippocampus. This study identifies the dorsal hippocampus as an important brain region for the expression of social fear and the NPY and serotonergic systems as possible targets in the treatment of SAD.

We confirm previous studies showing that SFC induces robust social fear in mice [23,36,43,46]. This social fear is expressed as reduced or absent investigation of unfamiliar conspecifics as well as intense aversive responses in the presence of conspecifics, such as attempt approaches and freezing.

IEGs are well-established markers of neuronal activation [33,34,47] and different IEGs such as cFos, Fosl2 and Arc show different temporal activation patterns after stress exposure [48,49,50]. Our IHC and immunofluorescence studies indicated lower cFos immunoreactivity in the dorsal hippocampus but not ventral hippocampus and no changes in cFos immunoreactivity in the PVN of the hypothalamus of SFC+ mice 2 h after the assessment of social fear. Within the dorsal hippocampus, the cFos immunoreactivity was reduced in the gcl of the DG but not in the pcl of the CA3 and CA1 regions, suggesting that the gcl of the DG of the dorsal hippocampus is involved in the expression of the SFC-induced social fear. An involvement of additional brain regions is highly likely given that the regulation of fear responses is dependent on the coordinated activity of the medial prefrontal cortex (mPFC), amygdala and hippocampus [51]. In highly simplified terms, the BLA receives sensory information from the thalamus and sensory cortex, as well as context-related information from the hippocampus, and projects to the central amygdala (CeA) that mediates the fear responses through projections to the hypothalamus and brainstem. The mPFC exerts top-down control over the amygdala via its prelimbic (PL) and infralimbic (IL) subdivisions to regulate appropriate behavioral responses, i.e., expression or suppression of fear, respectively [51,52]. Future studies will therefore investigate cFos immunoreactivity in other brain regions of the fear circuitry in SFC+ mice, such as the BLA, CeA, IL and PL cortex. In support of the involvement of additional brain regions in the expression of social fear, studies investigating stress-, traumata- and pharmacological-induced social avoidance described increased cFos immunoreactivity in the CeA and BLA [53,54,55] and reduced cFos and zif268 immunoreactivity in the mPFC [56]. A hyperactive amygdala coupled with a hypoactive mPFC and hippocampus may therefore lead to the increased expression of social fear observed in these studies and in our study.

Mice and rat studies investigating the effect of social interaction on neuronal activity in the hippocampus revealed no significant increase in cFos immunoreactivity in the DG and CA1, but an increase in the BLA, CeA, medial amygdala (MeA), bed nucleus of the stria terminalis (BNST) and CA3 [57,58,59,60]. This suggests that the reduced social investigation alone may not be the trigger for our findings of reduced cFos immunoreactivity in the DG of SFC+ mice and might indicate that an inhibitory mechanism could be involved in the memory processing of stress-related events and thus social fear expression. In support of this hypothesis, rats exposed to 10 min of immobilization stress showed a reduced cFos immunoreactivity in the gcl of the DG of the dorsal hippocampus [61]. A further study indicated that a reduction in cFos immunoreactivity after reoccurring negative stimuli could be part of a neuronal learning mechanism mediated by the IEG [62].

An intriguing question that arises at this point is whether the neuronal activity was reduced in the dorsal hippocampus of SFC+ mice or whether it was rather increased in SFC− mice. Given that social interaction did not alter cFos immunoreactivity in the hippocampus [57,58,59,60], it is highly unlikely that the cFos immunoreactivity was increased in SFC− mice due to greater interaction with the social stimulus. Similarly, a possible higher locomotor activity that might be expected in SFC− mice is unlikely to have increased cFos immunoreactivity in the dorsal hippocampus, as an increased locomotor activity was not associated with increased cFos levels in mice [63]. Besides, we found similar locomotor activity levels between SFC− and SFC+ mice. Although SFC− mice moved around the social stimulus more, corresponding to their increased social investigation, and SFC+ mice moved more at the opposite side of the cage, corresponding to their socially fearful and avoidant phenotype, these differences in locomotor patterns, but not in locomotor activity per se, are unlikely to have affected the cFos immunoreactivity.

Given that SFC+ mice showed a reduced cFos immunoreactivity in the dorsal and not in the ventral hippocampus, the gene expression study was performed only in the dorsal hippocampus. The reduced number and lower density of cFos-ir cells found in the gcl of the DG of the dorsal hippocampus in SFC+ mice was not replicated by decreased cFos mRNA levels. The cFos and Fosl2 mRNA levels were decreased in SFC+ mice in the pcl of CA3 and CA1 but not in the gcl of the DG. These differences might be explained by a different temporal activation pattern of investigated IEGs as well as the time-shifted activations of mRNA and protein. When investigating cFos mRNA levels, our time-point (2 h after social exposure being an optimal time-point for investigating the cFos protein [34]) captured the downward trend of cFos mRNA levels as it rapidly accumulates after stimulation and reaches its peak 30 min after neuronal activation [34]. In contrast, mRNA expression of Fosl2 is highest in the timespan of 1–2 h post stimulus [48]. As the Fosl2 gene is closely related to the cFos gene and shares a similar genetic structure, the Fosl2 protein likely fulfils a similar function as a transcriptional factor and therefore poses an alternative for a delayed IEG [64]. Furthermore, different methodical approaches might also explain the differences in cFos expression and number of cFos-positive cells. With IHC, we detect cells accumulating cFos exceeding a certain threshold, whereas extracting whole sub-regions/cell layers with laser capture microdissection leads to highly heterogeneous populations of cells containing cFos at various levels.

Our results, showing decreased cFos and Fosl2 mRNA levels in the pcl of CA3 and CA1 of the dorsal hippocampus of SFC+ mice, support previous studies using pharmacological induction of social fear and avoidance in rodents. As such, morphine administration reduced social interaction and cFos mRNA expression in the PFC of rats [65]. Rats and mice prenatally exposed to valproic acid developed an autistic-like phenotype characterized by social avoidance and impaired social interaction. These rats and mice also showed reduced cFos expression in the PFC and the CA3 and CA1 regions of the hippocampus [65,66], supporting our findings. On the other hand, autistic BTBR T(+)Itpr3(tf)/J (BTBR) mice show a strong increase in cFos expression in the CA3 but not CA1 and DG regions of the hippocampus and a strong activation of periaqueductal regions related to defensiveness after 10 min of free interaction with another mouse [67]. This study suggests that unavoidable social interactions are highly aversive for autistic BTBR mice and apparently contradicts our findings. However, besides differences in the pathology of autism and social fear, the controllability of the social encounter may play an important role. For example, cFos expression within the CA3 seems to increase after unavoidable social interaction when BTBR mice are “forced” to interact with a healthy C57Bl6 mouse [67], whereas cFos expression decreases in the CA3 and CA1 regions when the feared social contact can be avoided [65,66], like in our study. The avoidance of the feared social contact and thus a certain controllability of the social encounter can be obtained by enclosing the social stimulus so that the experimental mouse initiates all social contact. In support of this hypothesis, exposure to uncontrollable fear-inducing stimuli was shown to increase cFos expression in several brain regions, including the hippocampus, PFC and amygdala, in studies using contextual and auditory fear conditioning paradigms [68,69,70,71].

In addition to the dorsal hippocampus, we also investigated the mRNA level of IEGs in other brain regions known to be involved in social behavior or fear-related behavior, such as the septum, PVN, BLA and DR, and did not observe any changes in SFC+ mice, suggesting that these brain regions might not be involved in the expression of social fear. Given that the CeA mediates the fear responses through projections to the hypothalamus and brainstem [51,52], future studies will investigate mRNA level changes of IEGs in other brain regions in SFC+ mice, such as the CeA and periaqueductal regions that coordinate defensive responses and fear-evoked freezing responses [72,73].

Apart from IEG gene expression levels, we investigated expression levels of genes related to various neurotransmitter systems known to modulate social behavior and fear, including the serotonin system, the GABAergic system and the AVP, OXT and NPY systems [28,29,30,31]. We found brain-region-dependent differences between SFC+ and SFC− mice in the expression of NPY system-related genes such as *Npy* and its two analyzed receptors *Npyr1* and *Npyr2*. The strongest effects were found at the level of the septum, where *Npyr2* expression was increased in SFC+ mice. SFC+ mice also showed a tendency towards a decreased *Npy* expression in the DG, an increased expression of *Npyr1* in the BLA and PVN, and an increased *Npyr2* expression in the BLA. The NPY system is involved in the modulation of stress, where it displays anxiolytic and fear-reducing characteristics [74]. It was previously shown that i.c.v. administration of NPY reduced the expression of SFC-induced social fear [36] by simultaneously activating the postsynaptic NPYR1 and presynaptic NPYR2. Local manipulations revealed that these effects were mediated via NPY1R in the CeA and NPY2R in the dorsolateral septum [37], further supporting the important role of the NPY2R within the septum in the expression of social fear. These effects are not surprising given that the septum plays a critical role in the regulation of emotional behaviors, social behaviors and fear-related behaviors [75] and expresses high levels of *Npyr2* [76]. We could also show that the expression of *Npy*, *Npyr1* and *Npyr2* genes was not altered in SFC+ mice in the DG, CA3 and CA1 regions of the hippocampus, suggesting that the hippocampal NPY system is not involved in the expression of social fear. Consistent with these results, administration of NPY within the dorsal hippocampus did not alter the expression of social fear [77].

By performing qPCR with primer pairs specific for three serotonin receptor genes, we were able to show an increased *Htr2a* expression in the PVN of SFC+ mice. A trend towards higher expression levels of *Htr1a* and *Htr2c* in SFC+ mice was found in the PVN and septum, respectively. In support of a role of the serotonergic system in the expression of social fear, chronic administration of the SSRI paroxetine and of the tricyclic antidepressant amitriptyline, that also increases the serotonergic neurotransmission by blocking the serotonin transporter at presynaptic terminals, was shown to reduce the expression of SFC-induced social fear [23,25]. Previous studies investigating the role of HTR2A in the modulation of stress responses [78,79,80] and fear conditioning support our result of higher *Htr2a* expression in the PVN in response to social fear expression. Administration of an HTR2A agonist increased avoidance response after conditioned avoidance behavior [81], suggesting that upregulation of the HTR2A system might stimulate active stress coping mechanisms [82].

Evaluation of the expression of adult neurogenesis-related genes (*Dcx* and *Mcm2*), of genes related to the GABAergic system (*Gad1*), AVP system (*Avp*, *Avpr1a*) and OXT system (*Oxt*, *Oxtr*), did not show any alterations in SFC+ mice. This suggests that these neurotransmitter and neuropeptide systems might not be related to the expression of social fear, at least not at the level of the investigated brain regions. A lack of involvement of the AVP system in social fear was previously suggested by a pharmacological study showing that i.c.v. administration of AVP did not reduce the expression of SFC-induced social fear [35]. However, the lack of effects on the OXT system, especially at the level of the septum, are quite surprising given that i.c.v. administration of OXT reduced the expression of SFC-induced social fear, an effect that was mediated via OXTR within the dorsolateral septum [35,45]. Furthermore, social fear was accompanied by an increased OXTR binding in the dorsolateral septum that normalized after successful extinction of social fear [35], apparently contradicting our findings. Besides time-shifted activations of mRNA and protein, these results might point to different functions of the OXT system. As such, the OXT system seems to play an important role during social fear learning and extinction of social fear [35,45], but not during a short exposure to a social stimulus like in our study. In support of this hypothesis, OXT was released within the dorsolateral septum, starting approximately 30 min after exposure to social stimuli and not during the first 30 min of social contact [35], indicating that the 5 min exposure to the social stimulus in our study might not be enough to induce changes in *Oxtr* gene expression. Additionally, our study assessed *Oxtr* gene expression in the entire septum, whereas changes in OXTR binding were only observed in the dorsolateral septum but not in the ventrolateral septum [35]. It would therefore be interesting to investigate *Oxtr* gene expression separately for the dorsolateral and ventrolateral septum.

Limitations of the study: apart from the investigation of additional brain regions that would allow for a better understanding of the mechanisms involved in the expression of social fear, the study of female mice would determine whether these findings can be extrapolated to females, especially because SAD is more prevalent in women [83].

In conclusion, our study identifies the dorsal hippocampus as an important brain region for the expression of social fear and the NPY and serotonergic systems as possible targets in the treatment of SAD.

## 4. Materials and Methods

### 4.1. Animals

Male C57BL/6J mice (Charles River, Sulzfeld, Germany, 8 weeks of age) were individually housed for three days before experiments started and remained single-housed throughout the experiment. Mice were kept under standard laboratory conditions (12:12 light/dark cycle, lights on at 07:00 h, 22 °C, 60% humidity, food and water ad libitum). Experiments were performed during the light phase between 09:00 and 14:00 in accordance with the Guide for the Care and Use of Laboratory Animals of the Government of Unterfranken (approval code 55.2 DMS-2532-2-314, approval date 13 December 2016) and the guidelines of the NIH. All efforts were made to minimize animal suffering and to reduce the number of animals used.

### 4.2. Behavioral Experiment

#### 4.2.1. Social Fear Conditioning (SFC) Paradigm

To induce social fear, mice were conditioned during SFC using a computerized fear conditioning system (TSE System GmbH, Bad Homburg, Germany), as previously described [23,24,35,36,42,43,44,45]. Mice were placed in the conditioning chamber (45 cm × 22 cm × 40 cm) and, after a 30 s habituation period, an empty wire mesh cage (7 cm × 7 cm × 6 cm) was placed as a non-social stimulus near one of the short walls. After 3 min, the non-social stimulus was replaced by an identical cage containing an unfamiliar age- and sex-matched mouse. SFC− mice were allowed to investigate this social stimulus for 3 min, whereas SFC+ mice were given a 1 s mild electric foot shock (0.7 mA) each time they investigated, i.e., made direct contact with the social stimulus (Figure 1). Mice received between two and three foot shocks with a variable inter-shock interval, depending on when direct social contact was made. The number of foot shocks was assessed as a measure of distress and of social fear learning. Mice were returned to their home cage when no further social contact was made for 2 min (average duration of SFC was approximately 10 min). The time that the mice spent investigating, i.e., directly sniffing, the non-social stimulus as a pre-conditioning measure of non-social anxiety was analyzed.

#### 4.2.2. Social Fear Assessment

The SFC-induced social fear was assessed one day after SFC (Figure 1). Mice were exposed in their home cage to a cage containing an unfamiliar mouse (i.e., social stimulus; different from the social stimulus used on day 1 during SFC) for 5 min, to assess social investigation as a parameter of social fear. The stimulus was placed near a short wall of the home cage. The test was recorded and analyzed using JWatcher (V 1.0, Macquarie University, Sydney, Australia and UCLA, Los Angeles, CA, USA). Social investigation was defined as direct sniffing of the cage and/or of the social stimulus inside of the cage. The number of attempt approaches was counted and the time that the mice spent freezing was analyzed as additional fear responses. Attempt approaches are behavioral fear responses reflecting risk assessment where mice reach out to investigate the feared stimulus (i.e., social stimulus), without going through with an investigation, whereas freezing is a fear response characterized by cessation of all movement except for that required for respiration [23,43].

The locomotor activity during social fear assessment was also analyzed. For this, the surface of the home cage (35 cm × 19 cm) was divided in 15 imaginary rectangles of 7 cm × 6.3 cm each. The social stimulus was placed on one rectangle at the short wall of the home cage. The five rectangles around the social stimulus were defined as proximal rectangles, whereas the other nine rectangles were defined as distal rectangles. The number of line crossings (i.e., proximal, distal and total) was counted as a parameter of locomotor activity.

### 4.3. Tissue Preparation

Two hours after exposure to the social stimulus during social fear assessment, brains were collected. The brains used for immunohistochemistry (IHC)/immunofluorescence staining (i.e., cohort 1) were fixed by immersion in 4% PFA (dissolved in 0.1 M PBS, pH 7.5) for 48 h and then cryoprotected using 10% and 20% sucrose (dissolved in 0.1 M PBS, pH 7.5) for 24 h, consecutively. After cryoprotection, these brains were snap-frozen in pre-cooled isopentane and stored at −80 °C. The brains used for the gene expression analysis (i.e., cohort 2) were directly snap-frozen in pre-cooled isopentane and stored at −80 °C until further processing.

#### 4.3.1. Immunohistochemistry

Coronal sections of the brains used for the IHC study (cohort 1) were cut at 30 µm almost throughout the whole brain using a Leica CM1950 freezing microtome. These sections were transferred to HistoBond^®^+ adhesion slides (Marienfeld, Lauda-Königshofen, Germany) generating a one-in-sixth series, meaning that every 6th section was transferred to the very same slide, to the same series of sections. This resulted in six series of sections, and each series comprising sections with a distance of 180 µm from one section to the next. The tissue sections of one series of sections was used for single IHC staining with the cFos antibody and the tissue sections of another series of sections was used for double immunofluorescence labelling of cFos and AVP.

##### Single Immunohistochemistry Staining of cFos

Sections were air dried for 45 min and washed with PBS. They were treated with 0.6% H_2_O_2_ in PBS for 30 min. Antigen retrieval was performed using a citrate buffer (10 mM sodium citrate, 0.05% TWEEN 20, pH 6.0) for 20 min at 80 °C in a water bath. Sections were blocked with 5% normal goat serum (NGS) in PBS for 1.5 h. Incubation with the polyclonal primary antibody rabbit anti-cFos (1:750, Proteintech, Manchester, UK, #26192-1-AP) in 10% NGS was performed overnight at 4 °C. On the second day, sections were incubated for 30 min with the corresponding Histofine Simple Stain MAX PO detection system (rabbit, Nichirei, Japan). For visualization, DAB Peroxidase substrate (diluted 1:10 in peroxidase buffer, Vector Laboratories, Burlingame, CA, USA) was applied onto the sections for 10 min. Sections were dehydrated using ascending alcohol series and covered in Vitro-Clud (R. Langenbrinck GmBH, Emmendingen, Germany) for preservation.

##### Immunofluorescence Double Staining of cFos and Vasopressin

Sections were air dried for 45 min and washed with TBS. Antigen retrieval was performed using a citrate buffer (10 mM sodium citrate, pH 6.0) for 20 min at 80 °C in a water bath. Sections were blocked with 10% normal horse serum (NHS) and 1% Triton X-100 in TBS for 1 h. Incubation with the primary antibodies polyclonal rabbit anti-cFos (1:500, Proteintech, Manchester, UK) and monoclonal mouse anti-vasopressin (1:100, Santa Cruz, CA, USA, #sc-390723) in 5% NHS was applied over two nights at 4 °C. On the third day, sections were washed with PBS and incubated with the corresponding secondary antibodies donkey anti-rabbit Alexa Fluor 488 (1:400, Thermo Fisher Scientific, Waltham, MA, USA, #A-21206) and donkey anti-mouse Alexa Fluor 647 (1:400, Thermo Fisher Scientific, Waltham, MA, USA, #A-31571) for 1.5 h. Sections were again washed with TBS and covered in Fluoro-Gel (Electron Microscopy Sciences) for preservation. The AVP antibody was used as a form of counterstaining to determine the precise area of the PVN.

##### Quantification of Immunolabeled Cells

Light microscope images were captured using an Olympus BX51 microscope with 4x and 10x PLAN N air objectives using an MBF Bioscience CX ×9000 camera. The Stereo Investigator^®^ software from MBF Bioscience (Williston, ND, USA, software version 2018) was used to quantify cFos-ir cells, applying an unbiased stereology procedure. In more detail, cFos-ir cells were counted using a special variant of the optical fractionator method with counting frame and sampling grid of 150 by 150 μm size with ratio 1:1. We analyzed all counting frames in the already traced dorsal and ventral hippocampal regions [i.e., gcl of the DG, pcl of the CA3 and CA1] and, in consequence, counted all cFos-ir cells in the areas of interest of a tissue section. Systematic random sampling was not recommended as the number of cFos-ir cells in these layers of interest was not high enough. The first section to be analyzed was randomly selected and then all sections of one series of sections (see Section 4.3.1) was used for quantification, meaning that every 6th succeeding section throughout the dorsal–ventral axis of the hippocampus was analyzed. For quantitative evaluation, a fictive coronal separation plane along the dorsal–ventral axis of the hippocampus was used to divide the data obtained from sections of the dorsal and ventral hippocampus, as described by Karabeg and coworkers [84]. The first section, in which the corpus callosum no longer connects the two hemispheres (approx. interaural level 1.34 mm [85]) was declared as the first section of the ventral part of the hippocampus. All sections before that point were considered to hold the dorsal part of the hippocampus, and all sections after that point hold the ventral part of the hippocampus [84].

Fluorescence images of cFos/AVP double stained cells in the hypothalamus were captured using an Olympus FluoView FV1000 Confocal microscope with a 10x UPlanAPO 0.40 N.A. objective. Cell count and area size was measured using ImageJ version 1.53c (Fiji) [86].

#### 4.3.2. Gene Expression Analysis

##### Laser Capture Microdissection

The brains used for the gene expression analysis (cohort 2) were sectioned to 20 µm in a Leica CM1950 freezing microtome at −20 °C and the tissue sections were mounted on polyethylene naphthalate (PEN) membrane slides 2.0 µm (Leica, Wetzlar, Germany). Mounted tissue sections were fixed in 75% EtOH (diluted in DEPC-treated water) for 2 min, stained with 0.25% cresyl violet and then processed through an ascending ethanol series (75%, 90%, 100% ethanol for 20 s each) for decoloring. After an additional incubation for 1 min in 100% ethanol, the slides were air dried in 50 mL tubes with Silica Gel Orange (Roth, Karlsruhe, Germany), and the tissue was dissected using a Leica LMD6 laser microdissection system, which allowed the precise extraction of specific brain regions up to small sub-regions and cell layers. Brain regions of interest were the gcl of the DG, the pcl of CA3 and CA1 of the dorsal hippocampus (interaural level 2.86–1.26 mm), the septum (interaural level 4.98–3.82 mm), the BLA (interaural level 2.74–1.74 mm), the PVN (interaural level 3.22–2.58 mm) and the DR (interaural level −0.36–−1.04 mm) [84]. RNA was extracted from the dissected tissue using the miRNeasy Micro Kit (Qiagen, Hilden, Germany) and stored at −80 °C until further use.

##### Quantitative Real-Time PCR (qPCR)

Primer sequences were chosen using the NCBI BLAST tool (Table S5). Primers were purchased from Sigma-Aldrich (St. Louis, MO, USA). To perform the qPCR reactions, the cDNA stock solution was further diluted using water to a 1:20 working solution. Per sample, 0.5 μL forward and reverse primer (5 μM each) per sample were mixed with 5μL SYBR™ Select Master Mix (Thermo Fisher Scientific, Waltham, MA, USA) and 4 μL cDNA to a total volume of 10 μL. A 384-well plate in a CFX384 real-time PCR detection system (Bio-Rad) was used for the reactions. LinRegPCR program (version 2017) [87] was used to calculate mean PCR efficiencies. Evaluation of expression, normalization and calculation of relative gene expression results were obtained with qBase+ software (Biogazelle, Ghent, Belgium).

### 4.4. Statistical Analysis

All data were analyzed using SPSS Statistics 26 (IBM Corporation, NY, USA) (Tables S1–S4), and GraphPad Prism software version 8 (GraphPad Software, La Jolla, CA, USA) was used for data graphing. Data sets were visually inspected via boxplots to examine symmetry, skew, variance and outliers. Additionally, the Levene’s test for homogeneity of variances and a Shapiro–Wilk test of normality were performed. Statistical analysis was performed using either a two-sample t-test or a Mann–Whitney U test. The Mann–Whitney U test was applied when homogeneity of the variance and/or the normal distribution was not given. Statistical significance was set at *p* < 0.05. Significance levels are indicated as * *p* < 0.05, ** *p* < 0.01 and *** *p* < 0.001, and *p*-values between *p* > 0.05 and *p* < 0.1 were defined as a trend and are indicated with #.

## Data Availability

The datasets generated during the current study are available from the corresponding author on request.

## Supplementary Materials

The following supporting information can be downloaded at: https://www.mdpi.com/article/10.3390/ijms23158228/s1.
