# Supplementary material for: Social Fear Affects Limbic System Neuronal Activity and Gene Expression
Source: Int J Mol Sci. 2022 Jul 26;23(15):8228. doi: 10.3390/ijms23158228 (PMC9367789; doi:10.3390/ijms23158228)
Supplement: Supplementary file 1 [file ijms-23-08228-s001.zip › ijms-1837326-supplementary.pdf]

International Journal of *Molecular Sciences*

MDPI

Supplementary Materials

Table S1. Descriptive and explorative statistics of behavioral data. Mice were exposed to social fear conditioning (SFC) on day 1 and the SFC-induced social fear was assessed on day 2 in unconditioned control mice (SFC-) and in conditioned socially fearful mice (SFC+). The percentage of investigation time towards the non-social as well as the social stimulus is presented for cohort 1 (immunohistochemistry study) and cohort 2 (gene expression study). During social fear assessment on day 2, we analyzed the number of attempt approaches and the percentage of freezing as additional indicators of fear, as well as the number of proximal, distal and total line crossings in the home cage as indicators of locomotor activity. Statistical analysis was performed using either a two-sample t-test or a Mann-Whitney-U test. The Levene Test for homogeneity of variances and the Shapiro-Wilk Test of normality were performed. The Mann-Whitney-U Test was applied when homogeneity of the variance and/or the normal distribution was violated. Data represent means ± SEM. Bold entries highlight a significant p-value.

**Behavioral Results**								
		**Mean (±SEM) of different treatments**		Two-sample t-test p-value	Mann-Whitney-U Test p-value	Levene-Test p-value	Shapiro-Wilk Test p-value	
		**SFC-**	SFC+	SFC+ vs. SFC-	SFC+ vs. SFC-		SFC-	SFC+
**Cohort 1 (immunohistochemistry study)**								
**SFC (day 1)**	**Non-social investigation (%)**	13.5 (±1.62)	11.4 (±1.13)	0.316		0.480	0.303	0.536
	**Social investigation (%)**	34.69 (±2.14)						
**Social fear assessment (day 2)**	**Social investigation (%)**	28.5 (±1.73)	0.35 (±0.07)		**<0.001**	**0.001**	0.290	0.979
	**Attempt approaches (n)**	0	4.1 (±0.89)					
	**Freezing (%)**	0	8.01 (±1.28)					
	**Proximal line crossings (n)**	50,6 (±5.53)	10.4 (±1.92)		**<0.001**	**0.042**	0.694	0.160
	**Distal line crossings (n)**	68.3 (±5.23)	125.0 (±13.34)		**0.001**	**<0.001**	0.806	**0.043**
	**Total line crossings (n)**	118.9 (±7.43)	135.4 (± 14.6)		0.345	**0.005**	0.953	0.103
**Cohort 2 (*gene expression study)***								
**SFC (day 1)**	**Non-social investigation (%)**	10.4 (±1.49)	11.1 (±1.43)		0.543	**0.001**	0.023	0.998
	**Social investigation (%)**	26.8 (±2.52)						
**Social fear assessment (day 2)**	**Social investigation (%)**	27.5 (±2.46)	0.145 (±0.55)		**<0.001**	0.921	0.305	**0.019**
	**Attempt approaches (n)**	0	4.7 (±0.68)					
	**Freezing (%)**	0	9.18 (±1.72)					

**Proximal line crossings (n)**	50.6 (±5.19)	11.4 (± 2.29)	**<0.001**	0.067	0.685	0.541
**Distal line crossings (n)**	74.5 (± 4.03)	109.9 (±8.20)	**0.001**	0.068	0.490	0.203
**Total line crossings (n)**	125.1 (±7.28)	121.3 (±9.51)	**0.755**	0.569	0.631	0.198

Table S2. Descriptive and explorative statistics of the quantitatively evaluated cFos-positive cells in the dorsal (d) and ventral (v) hippocampus - an immunohistochemical study. Target regions include the granule cell layer of the dentate gyrus (DG), the pyramidal cell layer of cornu ammonis area 3 (CA3) and 1 (CA1). Statistical analysis was performed using either a two-sample t-test or a Mann-Whitney-U test. The Levene Test for homogeneity of variances and the Shapiro-Wilk Test of normality were performed. The Mann-Whitney-U Test was applied when homogeneity of the variance and/or the normal distribution was violated. Data represent means ± SEM. Bold entries highlight a significant p-value. Entries in italics indicate a trend towards a significant difference. SFC-, unconditioned control mice; SFC+, conditioned socially fearful mice.

**cFos immunohistochemistry**							
**Region**	Mean (±SEM) of different treatments		Two-sample t-test p-value	Mann-Whitney-U Test p-value	Levene-Test p-value	Shapiro-Wilk Test p-value	
	SFC-	SFC+	SFC+ vs. SFC-	SFC+ vs. SFC-		SFC-	SFC+
**mean cFos-ir cells**							
**dDG**	17.92 (±1.74)	11.66 (±0.88)	**0.004**		0.130	0.887	0.110
**dCA3**	6.88 (±0.85)	7.65 (±1.15)	0.606		0.105	0.481	0.400
**dCA1**	0.2 (±0.07)	0.31 (±0.08)		0.285	0.807	**0.047**	*0.084*
**vDG**	14.4 (±1.62)	12.1 (±0.88)	0.217		0.83	0.488	0.355
**vCA3**	17.64 (±1.82)	20.14 (±1.68)	0.324		0.969	0.876	0.700
**vCA1**	0.49 (±0.32)	0.46 (±0.2)		0.794	0.694	**0.001**	**0.001**
**mean area (mm²)**							
**dDG**	0.162 (±0.006)	0.168 (±0.005)		0.497	0.482	**0.023**	0.132
**dCA3**	0.119 (±0.004)	0.124 (±0.004)	0.407		0.855	0.794	0.205
**dCA1**	0.071 (±0.003)	0.083 (±0.004)		*0.053*	*0.090*	**0.008**	0.929
**vDG**	0.159 (±0.006)	0.168 (±0.004)	0.215		0.284	0.515	0.710
**vCA3**	0.319 (±0.017)	0.337 (±0.015)	0.436		0.730	0.670	*0.066*
**vCA1**	0.010 (±0.009)	0.111 (±0.007)	0.341		0.740	0.383	0.495
**mean cell density (cFos-ir cells/mm³) × 10⁻³**							
**dDG**	4.954 (±0.313)	3.147 (±0.249)		**0.004**	*0.057*	0.660	**0.037**
**dCA3**	2.605 (±0.235)	2.756 (±0.388)	0.769		0.470	0.525	0.890

**dCA1**	0.128 (±0.043)	0.165 (±0.044)		0.367	0.985	**0.026**	0.144
**vDG**	4.125 (±0.526)	3.263 (±0.289)	0.158		0.71	0.107	0.314
**vCA3**	2.500 (±0.235)	2.720 (±0.222)	0.505		0.840	0.545	0.984
**vCA1**	0.176 (±0.010)	0.219 (±0.011)		0.923	0.531	**0.001**	**0.001**

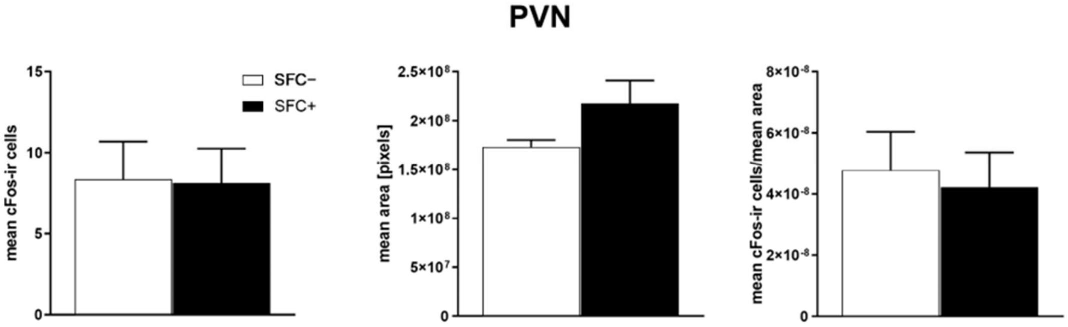

Figure S1. Quantitative evaluation of cFos-immunoreactive cells in the paraventricular nucleus (PVN). (**a**) Mean number of cFos immunoreactive (cFos-ir) cells in examined sections. (**b**) Mean area of investigated layers in pixels. (**c**) Mean cell density (mean number of cFos-ir cells/mean area). SFC-, unconditioned control mice (n=9); SFC+, conditioned socially fearful mice (n=10). Statistical analysis was performed using a two-sample comparison test. Data represent means + SEM.

Table S3. Descriptive and explorative statistics of the quantitatively evaluated cFos-positive cells in the paraventricular nucleus (PVN) of the hypothalamus - an immunofluorescence study. Statistical analysis was performed using either a two-sample t-test or a Mann-Whitney-U test. The Levene Test for homogeneity of variances and the Shapiro-Wilk Test of normality were performed. The Mann-Whitney-U Test was applied when homogeneity of the variance and/or the normal distribution was violated. Data represent means ± SEM. Bold entries highlight a significant p-value. Entries in italics indicate a trend towards a significant difference. SFC-, unconditioned control mice; SFC+, conditioned socially fearful mice.

**cFos immunofluorescence**							
**Region**	Mean (±SEM) of different treatments		Two-sample t-test p-value	Mann-Whitney-U Test p-value	Levene-Test p-value	Shapiro-Wilk Test p-value	
	SFC-	SFC+	SFC+ vs. SFC-	SFC+ vs. SFC-		SFC-	SFC+
**mean cFos-ir cells**							
**PVN**	8.352 (±2.339)	8.103 (±2.156)		0.579	0.524	**0.006**	**0.015**
**mean area (pixels)**							
**PVN**	173283136 (±7077120)	217545735 (±23499393)		0.105	**0.018**	0.234	0.625
**mean cell density (cFos-ir cells/pixels) $\times 10^{-3}$**							
**PVN**	4.776E-8 (±1.261E-8)	4.22E-8 (±1.144E-8)		0.912	0.512	**0.010**	*0.074*

Table S4. Descriptive and explorative statistics of quantitative real time-PCR data. Sown are the data for (**a**) the granule cell layer of the dentate gyrus, (**b**) the pyramidal cell layer of the cornu ammonis area 3 and (**c**) cornu ammonis area 1 of the dorsal hippocampus, (**d**) the septum, (**e**) the paraventricular nucleus of the hypothalamus, (**f**) the basolateral amygdala and (**g**) the dorsal raphe. Statistical analysis was performed using either a two-sample t-test or a Mann-Whitney-U test. The Levene Test for homogeneity of variances and the Shapiro-Wilk Test of normality were performed. The Mann-Whitney-U Test was applied when homogeneity of the variance and/or the normal distribution was violated. Data represent relative expression means ± SEM. Bold entries highlight a significant p-value. Entries in italics indicate a trend towards a significant difference. SFC-, unconditioned control mice; SFC+, conditioned socially fearful mice.

**Table S4a**				**Dentate gyrus**			
**Gene**	Mean (±SEM) of different treatments		Two-sample t-test p-value	Mann-Whitney-U Test p-value	Levene-Test p-value	Shapiro-Wilk Test p-value	
	SFC-	SFC+	SFC+ vs. SFC-	SFC+ vs. SFC-		SFC-	SFC+
Arc	1.15 (±0.13)	1.06 (±0.21)	0.718		0.106	0.142	0.201
Avpr1a	0.95 (±0.07)	0.89 (±0.21)	0.646		0.506	0.341	0.490
cFos	1.2 (±0.18)	1.11 (±0.17)	0.715		0.731	0.142	0.131
Dcx	1.03 (±0.06)	1.03 (±0.09)		0.971	0.357	0.015	0.867
Fosl2	1.3 (±0.17)	1.06 (±0.20)	0.382		0.600	0.056	0.120
Gad1	1.02 (±0.06)	1.02 (±0.08)		0.739	0.008	0.297	0.692
Htr1a	1.07 (±0.07)	0.97 (±0.14)	0.516		0.120	0.350	0.239
Htr2a	1.02 (±0.04)	1.06 (±0.08)	0.682		**0.026**	0.619	0.345
Mcm2	0.97 (±0.05)	1.02 (±0.04)		1.000	**0.032**	0.094	0.518
Npy	1.07 (±0.05)	1.05 (±0.12)	0.241		0.63	0.758	0.696
Npyr1	1.08 (±0.05)	1.0 (±0.06)	0.374		0.523	0.380	0.855
Npyr2	1.03 (±0.04)	1.0 (±0.09)		0.364	**0.021**	**0.02**	0.499
Oxtr	1.09 (±0.13)	1.07 (±0.13)	0.897		0.797	0.357	0.237
**Table S4b**				**Cornu ammonis 3**			
**Gene**	Mean (±SEM) of different treatments		Two-sample t-test p-value	Mann-Whitney-U Test p-value	Levene-Test p-value	Shapiro-Wilk Test p-value	
	SFC-	SFC+	SFC+ vs. SFC-	SFC+ vs. SFC-		SFC-	SFC+
Arc	0.82 (±0.05)	0.95 (±0.07)	0.143		0.366	0.330	0.245
cFos	0.83 (±0.05)	1.14 (±0.08)	**0.006**		*0.068*	0.768	0.557
Fosl2	0.96	1.24	**0.037**		0.485	0.551	0.908

	(±0.08)	(±0.10)					
Gad1	0.95 (±0.02)	1.02 (±0.03)	0.075		0.212	0.910	0.679
Htr1a	1.09 (±0.07)	1.05 (±0.07)	0.591		0.308	0.779	0.883
Htr2a	1.0 (±0.03)	1.04 (±0.04)	0.472		*0.096*	0.376	0.667
Npy	1.0 (±0.04)	1.02 (±0.06)	0.799		*0.092*	0.204	0.653
Npyr1	0.97 (±0.03)	1.0 (±0.06)	0.712		0.204	0.285	0.487
Npyr2	1.01 (±0.03)	1.02 (±0.03)	0.881		0.367	0.412	0.133
Oxtr	1.07 (±0.03)	1.04 (±0.07)		0.631	0.045	0.638	0.461
**Table S4c**					**Cornu ammonis 1**		
**Gene**	Mean (±SEM) of different treatments		Two-sample t-test p-value	Mann-Whitney-U Test p-value	Levene-Test p-value	Shapiro-Wilk Test p-value	
	SFC-	SFC+	SFC+ vs. SFC-	SFC+ vs. SFC-		SFC-	SFC+
Arc	0.92 (±0.04)	0.89 (±0.06)	0.681		0.644	0.414	0.253
cFos	0.85 (±0.09)	0.68 (±0.07)	0.149		0.221	0.243	0.700
Fosl2	1.24 (±0.09)	0.97 (±0.07)	**0.032**		0.503	0.896	0.768
Gad1	1.04 (±0.04)	1.0 (±0.05)	0.626		0.510	0.847	*0.077*
Htr1a	1.09 (±0.06)	1.13 (±0.06)	0.650		0.677	0.981	0.407
Htr2a	1.06 (±0.05)	1.15 (±0.04)	0.164		0.567	0.477	0.288
Npy	0.96 (±0.04)	1.04 (±0.06)	0.262		*0.058*	*0.081*	0.78
Npyr1	0.96 (±0.04)	1.02 (±0.07)	0.475		*0.082*	0.921	0.959
Npyr2	0.96 (±0.04)	1.03 (±0.11)	0.248		0.971	0.517	0.797
Oxtr	1.21 (±0.16)	0.98 (±0.18)		0.280	0.912	**0.038**	**0.041**
**Table S4d**					**Septum**		
**Gene**	Mean (±SEM) of different treatments		Two-sample t-test p-value	Mann-Whitney-U Test p-value	Levene-Test p-value	Shapiro-Wilk Test p-value	
	SFC-	SFC+	SFC vs. SFC-	SFC+ vs. SFC-		SFC-	SFC+
Arc	0.99 (±0.07)	0.91 (±0.05)	0.329		0.414	0.774	0.605
Avpr1a	0.91 (±0.07)	1.12 (±0.12)	0.163		0.106	0.379	0.503
cFos	0.82	0.82	0.962		0.812	0.827	0.355

	(±0.04)	(±0.04)					
Fosl2	1.19 (±0.11)	1.14 (±0.07)		0.853	0.200	0.491	**0.017**
Htr1a	0.98 (±0.03)	1.06 (±0.05)	0.219		0.281	0.873	0.059
Htr2a	1.09 (±0.03)	1.08 (±0.05)	0.882		0.312	0.973	0.934
Htr2c	0.96 (±0.02)	1.04 (±0.04)	*0.079*		*0.097*	*0.069*	0.709
Npy	1.18 (±0.11)	0.94 (±0.11)		0.130	0.578	**0.048**	0.065
Npyr1	1.08 (±0.07)	1.04 (±0.07)	0.702		0.613	0.692	0.147
Npyr2	0.93 (±0.06)	1.21 (±0.10)		**0.035**	0.222	**0.015**	0.915
Oxtr	1.01 (±0.04)	1.00 (±0.04)	0.901		0.525	0.348	0.176

**Table S4e**			**Paraventricular nucleus**				
**Gene**	Mean (±SEM) of different treatments		Two-sample t-Test p-value	Mann-Whitney-U Test p-value	Levene-Test p-value	Shapiro-Wilk Test p-value	
	SFC-	SFC+	SFC+ vs. SFC-	SFC+ vs. SFC-		SFC-	SFC+
Arc	1.07 (±0.14)	1.06 (±0.06)		0.436	0.146	**0.015**	0.486
Avp	1.09 (±0.17)	0.91 (±0.16)	0.456		0.769	0.248	0.483
Avpr1a	1.01 (±0.08)	1.06 (±0.11)	0.737		0.161	*0.061*	0.667
cFos	0.99 (±0.12)	1.09 (±0.07)	0.463		*0.054*	*0.059*	0.906
Fosl2	0.98 (±0.05)	1.05 (±0.11)		0.631	**0.017**	0.316	0.683
Htr1a	0.901 (±0.07)	1.09 (±0.09)		*0.089*	0.765	**0.023**	0.200
Htr2a	0.97 (±0.05)	1.16 (±0.11)		**0.023**	0.246	0.942	**0.045**
Htr2c	0.97 (±0.05)	1.02 (±0.04)		0.353	0.858	**0.031**	**0.045**
Npy	1.07 (±0.10)	0.93 (±0.09)	0.328		0.768	0.893	0.236
Npyr1	0.93 (±0.05)	1.13 (±0.09)	*0.064*		0.370	0.509	0.124
Npyr2	0.94 (±0.07)	1.04 (±0.11)	0.436		0.385	0.491	0.668
Oxt	0.97 (±0.15)	0.99 (±0.14)	0.922		0.558	0.114	*0.087*
Oxtr	1.00 (±0.07)	1.16 (±0.08)	0.188		0.958	0.284	0.198

**Table S4f**			**Basolateral amygdala**				

**Gene**	Mean (±SEM) of different treatments		Two-sample t-Test p-value	Mann-Whitney-U Test p-value	Levene-Test p-value	Shapiro-Wilk Test p-value	
	SFC-	SFC+	SFC+ vs. SFC-	SFC vs. SFC-		SFC-	SFC+
Arc	0.90 (±0.12)	0.85 (±0.06)		0.739	**0.005**	*0.065*	0.138
cFos	0.90 (±0.10)	0.95 (±0.13)		0.971	0.489	0.398	**0.017**
Fosl2	1.06 (±0.08)	1.06 (±0.11)	0.990		0.425	0.345	0.539
Htr1a	0.46 (±0.04)	0.48 (±0.03)	0.777		0.588	0.284	0.884
Htr2a	1.07 (±0.10)	0.98 (±0.06)	0.455		0.397	*0.087*	0.218
Htr2c	0.94 (±0.05)	1.01 (±0.05)	0.401		0.735	*0.076*	*0.071*
Npy	1.00 (±0.06)	0.97 (±0.09)		0.579	0.955	0.198	**0.008**
Npyr1	0.97 (±0.04)	1.10 (±0.05)	*0.069*		0.765	0.991	0.495
Npyr2	0.97 (±0.06)	1.18 (±0.08)	*0.074*		0.301	0.267	0.346
Oxtr	1.10 (±0.09)	1.03 (±0.07)	0.564		0.565	0.872	0.923

**Table S4g**				**Dorsal raphe**			
**Gene**	Mean (±SEM) of different treatments		Two-sample t-Test p-value	Mann-Whitney-U Test p-value	Levene-Test p-value	Shapiro-Wilk Test p-value	
	SFC-	SFC+	SFC+ vs. SFC-	SFC+ vs. SFC-		SFC-	SFC+
Arc	1.04 (±0.11)	1.00 (±0.08)	0.806		0.712	0.112	0.863
Avpr1a	1.18 (±0.10)	0.89 (±0.13)	0.107		0.170	0.241	0.266
cFos	1.00 (±0.07)	1.03 (±0.07)	0.764		0.921	0.152	0.637
Fosl2	1.07 (±0.09)	1.03 (±0.06)	0.712		0.156	0.805	*0.094*
Htr1a	1.04 (±0.05)	0.95 (±0.10)	0.425		*0.083*	0.825	0.332
Htr2a	0.94 (±0.15)	1.37 (±0.21)		0.113	**0.046**	*0.058*	*0.089*
Htr2c	0.99 (±0.04)	0.99 (±0.03)	0.795		0.654	0.858	0.201
Npy	1.45 (±0.23)	0.89 (±0.25)		*0.077*	0.631	0.857	**0.031**
Npyr1	1.09 (±0.07)	0.95 (±0.06)		0.222	0.625	**0.031**	0.779
Npyr2	1.08 (±0.08)	0.97 (±0.12)	0.472		0.115	0.420	0.462
Oxtr	1.05	0.95	0.442		0.472	0.482	0.400

	(±0.08)	(±0.08)					
Tph2	1.27<br>(±0.12)	1.10<br>(±0.20)	0.477		*0.077*	0.981	0.350

Table S5: List of primers used for the quantitative real time-PCR. F: Forward primer sequence; R: Reverse primer sequence. Manufactured by Sigma-Aldrich.

Symbol	Gene	Product [bp]	RefSeq.	Primer-Sequence
		Reference genes		
Actb	Actin beta	84	NM_007393.5	F: ATGTGGATCAGCAAGCAGGA<br>R: AGCTCAGTAACAGTCCGCCTA
B2m	Beta 2 microglobulin	126	NM_009735.3	F: ACCGTCTACTGGGATCGAGA<br>R: TGCTATTTCTTTCTGCGTGCAT
Gapdh	Glyceraldehyde 3-phosphate dehydrogenase	135	NM_008084	F: GTGATGGGTGTGAACCACGA<br>R: GGTCATGAGCCCTTCCACAA
Gdi2	GDP dissociation inhibitor beta	126	NM_008112	F: GTCAGAATTGGTTGGTTCTGTTC<br>R: AGCTCTTGGATCACACAATCG
Rplp0	60S acidic ribosomal protein P0	83	NM_007475	F: GAGGCCACACTGCTGAACAT<br>R: ATGCTGCCGTTGTCAAACAC
Target genes				
Arc	Activity-regulated cytoskeleton-associated protein	82	NM_018790	F: GGGTGAGCTGAAGCCACAAA<br>R: ACTGGTATGAATCACTGGGGG
Avp	Arginine vasopressin	125	NM_009732.2	F: CAAGAGGCGGCAAGAGGG<br>R: CACGAAGCAGCCCAGCTC
Avpr1a	Arginine vasopressin receptor 1a	114	NM_016847.2	F: CCTACATCCTCTGCTGGACAC<br>R: CCAGTAACGCCGTGATCGT
cFos	FBJ osteosarcoma oncogene	149	NM_010234.3	F: GGCAGAAGGGGCAAAGTAGAG<br>R: TCAAGTTGATCTGTCTCCGCTTG
Dcx	Neuronal migration protein doublecortin	98	NM_001110222	F: TTGGAAGCATGGATGAACTG<br>R: GTTGGGGTTGACATTCTTGG
Fosl2	Fos-like antigen 2	107	NM_008037.4	F: GGTTTCTACGGGGAAGAGCC<br>R: TCCAGGACATTGGGGTAGGT
Gad1	Glutamate decarboxylase 1	120	NM_008077.5	F: ATATTTTCTCCTGGGGGAGCC<br>R: GGTGAAGAGGACCAGTTTGGG
Htr1a	Serotonin 1A receptor	144	NM_008308.4	F: GATCTCGCTCACTTGGCTCA<br>R: AAAGCGCCGAAAGTGGAGTA
Htr2a	Serotonin 2A receptor	112	NM_172812.3	F: CCATAGCCGCTTCAACTCCA<br>R: CGAATCATCCTGTAGCCCGA
Htr2c	Serotonin 2C receptor	73	NM_008312	F: GCAATAATGGTGAACCTGGGC<br>R: ACTGCCAAACCAATAGGCCA
Npy	Neuropeptide Y	106	NM_023456.3	F:CAGATACTACTCCGCTCTGCGACAC<br>TACAT<br>R:TTCCTTCATTAAGAGGTCTGAAATCA<br>GTGTCT
Npyr1	Neuropeptide Y receptor 1	105	NM_010934	F: ATTTCGGCCCACTCTGCTTT<br>R: ACCTGTACTTACTGTCCCGGA
Npyr2	Neuropeptide Y receptor 2	77	NM_008731	F: CATCTGAGAAGGAACGCGCA<br>R: CTACCGGGCCCATCTTCAGA
Mcm2	Minichromosome maintenance complex component 2	146	NM_008564.2	F: GCAACTTTGTACTGGGGCCT<br>R: CTCCTGGATGCGGATACGTT
Oxt	Oxytocin	141	NM_011025	F: GAGGAGAACTACCTGCCTTCG<br>R: CGAGAAGGCAGACTCAGGGT

Oxtr	Oxytocin receptor	123	NM_001081147	F: ACGTCAATGCGCCCAAAGAA<br>R: GCACGAGTTCGTGGAAGAGAT
Tph2	Tryptophan hydroxylase 2	184	NM_173391	F: TGGGGATTTGATGCCTAGAACC<br>R: TGGGTTCTTTAGAGCATTTTTGTGT
